# The potential benefits of radiotherapy in elderly patients with early-stage triple-negative breast cancer

**DOI:** 10.3389/fmed.2024.1525425

**Published:** 2025-01-08

**Authors:** Zheng Xu, Chengdong Qin, Binxiao Cao, Pengcheng Ruan, Mianhao Zhang, Guidong Chen

**Affiliations:** ^1^Department of General Surgery, The People's Hospital of Fenghua Ningbo, Ningbo, China; ^2^Department of General Surgery, Zhejiang Cancer Hospital Ningbo Branch, Ningbo, China; ^3^Department of Breast Surgery, Zhejiang Cancer Hospital, Ningbo, China

**Keywords:** triple-negative breast cancer (TNBC), elderly patient, radiotherapy, propensity score matching (PSM), nomogram, SEER program

## Abstract

**Background:**

Breast cancer (BC) is the most common cancer in women in the U.S. and a leading cause of cancer-related deaths. The incidence rises with age, especially in women over 70. Older patients often face multiple comorbidities, complicating treatment decisions. This study will analyze the role of radiotherapy (RT) in early-stage triple-negative breast cancer (TNBC) among elderly patients using the SEER database to assess its impact on survival outcomes.

**Methods:**

The patients aged 70+ with T1-2N0-1M0 TNBC were selected from the SEER database (2010–2015) according to specific inclusion and exclusion criteria. Statistical analyses involved chi-square tests, propensity score matching (PSM), and Cox regression to identify risk factors. A nomogram was developed, and Kaplan-Meier analysis compared overall (OS) and breast cancer-specific survival (BCSS) across different groups.

**Results:**

A total of 3,024 elderly patients with early-stage TNBC were analyzed. After employing PSM to eliminate baseline differences, survival analysis indicated that the breast-conserving surgery (BCS) group could benefit from RT (OS, HR = 0.68, *p* < 0.001; BCSS, HR = 0.64, *p* = 0.001). Cox regression analysis on the non-RT cohort within the BCS group identified age, tumor grade, and T stage as independent risk factors. Subsequently, a nomogram was developed to stratify patients and found that RT significantly improved OS and BCSS in the intermediate-risk (OS, HR = 0.49, 95% CI = 0.34–0.71, *p* = 0.001; BCSS, HR = 0.40, 95% CI = 0.21–0.77, *p* = 0.018) and high-risk group (OS, HR = 0.67, 95% CI = 0.55–0.81, *p* < 0.001; BCSS, HR = 0.61, 95% CI = 0.45–0.83, *p* = 0.007), while showing no significant benefit in the low-risk group (all *p*-values > 0.05).

**Conclusion:**

RT significantly improves OS and BCSS in early-stage TNBC patients after BCS, particularly for intermediate to high-risk individuals, while low-risk patients may omit it.

## 1 Introduction

According to the 2023 Global Cancer Statistics, breast cancer (BC) has emerged as one of the most prevalent cancers among women in the United States and is the second leading cause of cancer-related deaths in women, following lung cancer (1). It is estimated that in 2023, more than 290,000 new cases of BC will be diagnosed, and over 40,000 deaths will occur due to BC (1). The incidence of BC correlates positively with age, with women aged over 70 years accounting for more than 30% of all diagnosed cases (2). Besides, another study shows that 45% of BC cases occur in women aged 65 and older, with 33% in those over 70. Yet, only 3% of women over 70 participate in clinical research. This may lead to significant discrepancies in the clinical management of this patient group (≥70's) (3, 4). Due to the lack of sufficient research, physicians may hesitate in treatment choices, potentially affecting the treatment outcomes and quality of life for elderly patients. Previous studies have shown that BC incidence has been rising by 1.0% annually for women in their 60's since 2004, and by 1.2% annually for women over 70's since 2005 (5). And although breast cancer mortality has generally declined, with a 58% reduction in the United States from 1975 to 2019, the decrease in mortality is smaller among elderly breast cancer patients compared to other age groups (6–8). Older patients often have multiple underlying health conditions and diminished physical function compared to other populations. Additionally, they experience a higher incidence of postoperative complications, which complicates clinical management (9). Furthermore, the inadequate representation of older adults in clinical trials has resulted in a dearth of prospective studies focused on breast cancer in this population. Consequently, the management of elderly patients in clinical practice increasingly prioritizes personalized treatment strategies (10).

Radiotherapy (RT) is a standard first-line treatment for breast cancer, used to eradicate residual cancer cells and minimize the risk of recurrence. Nonetheless, the necessity of RT for elderly BC patients continues to be a subject of considerable debate. Research indicates that the elderly population has a higher prevalence of hormone receptor-positive tumors, which are typically associated with reduced biological aggressiveness. Consequently, it is often advisable for treatment strategies to adopt a more conservative approach (11, 12). Certain studies indicate that the high prevalence of comorbidities and other factors contribute to elevated mortality rates in elderly patients. As a result, RT does not enhance survival in those with early-stage BC (13–16). On the other hand, numerous studies suggest that RT reduces local recurrence rate (LRR) in BC patients, while a conservative treatment approach for the elderly may worsen breast cancer-specific survival (BCSS) (17, 18). Furthermore, it is important to highlight that several prior studies have not adequately addressed molecular subtyping, which is a crucial determinant in shaping treatment strategies for BC patients. Triple-negative breast cancer (TNBC) is a subtype of BC distinguished by the lack of estrogen receptors (ER), progesterone receptors (PR), and human epidermal growth factor receptor 2 (HER2) in tumor cells (19). Due to the absence of relevant receptor markers, patients with TNBC do not respond to standard endocrine or HER2-targeted therapies, leading to a generally poor prognosis. According to previous study, TNBC patients were more likely to experience distant recurrence (HR = 2.6; 95% CI = 2.0–3.5; *p* < 0.0001) and mortality (HR = 3.2; 95% CI = 2.3–4.5; *p* < 0.001) within 5 years of diagnosis compared to those with other subtypes (20). Over 50% of patients experience recurrence within 3 to 5 years of diagnosis, and the median overall survival (OS) with current treatment approaches is just 10.2 months (19, 21).

Accurate selection of elderly patients (≥70's) for radiotherapy is essential. This study will use the Surveillance, Epidemiology, and End Results (SEER) database to analyze early-stage elderly patients with TNBC. The patients will be divided into RT and non-RT cohorts to assess the impact of RT on OS and BCSS. Additionally, we will construct a nomogram to evaluate individual patient scores and categorize them based on these scores, thereby aiding in the identification of the most appropriate candidates for RT.

## 2 Methods

### 2.1 Study population

We selected T1-2N0-1M0 TNBC patients over the age of 70 from the SEER^*^Stat program (version 8.4.4) for this study. Established in 1973, the SEER database is a crucial resource provided by the National Cancer Institute (NCI). Its primary aim is to offer publicly accessible epidemiological data to support cancer research, prevention, and control efforts. The database includes information from numerous states and regions, covering ~30% of the U.S. population, thereby ensuring the representativeness of the data (22).

**Figure 1:**
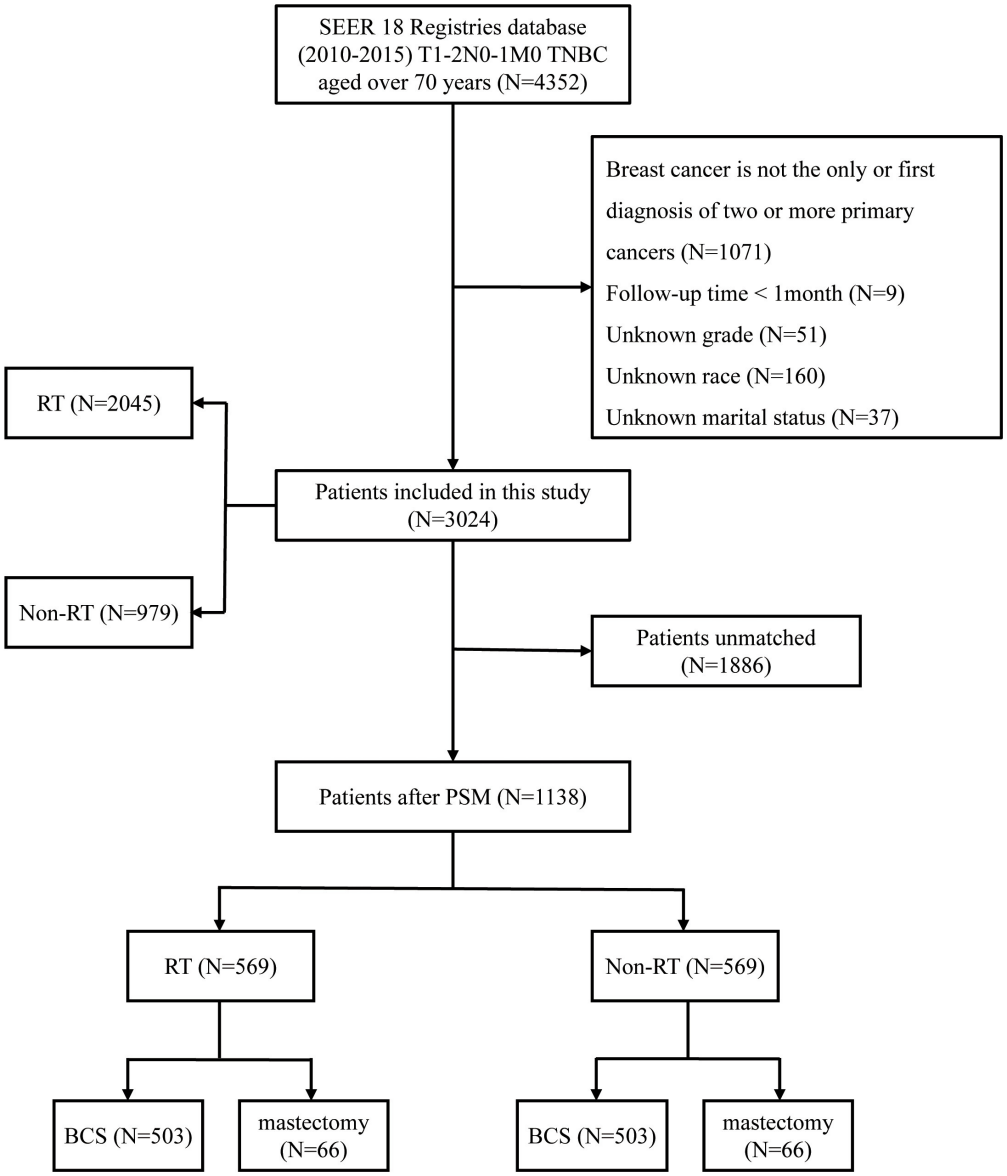
Flowchart of the study. TNBC, triple-negative breast cancer; RT, radiotherapy; PSM, propensity score matching; BCS, breast-conserving surgery.

The inclusion criteria for our study are as follows: 1. Diagnosis between 2010 and 2015, as the SEER database began collecting data on chemotherapy and radiotherapy after 2010; 2. Female patients aged 70 years or older; 3. Tumor staging classified as T1-2N1M0; 4. Molecular subtype identified as TNBC; 5. Surgical interventions comprising either modified radical mastectomy or breast-conserving surgery. The exclusion criteria are: 1. Cases where breast cancer is not the sole or initial diagnosis of two or more primary cancers; 2. Absence of critical information, including grade classification, survival months, race, and marital status; 3. Follow-up duration of < 1 month. Based on the aforementioned criteria, our study included a total of 3,024 patients. Based on whether patients received RT, the overall population was categorized into a RT group (*N* = 2,045) and a non-RT group (*N* = 979). The primary endpoints of this investigation are OS and BCSS. OS is defined as the time from disease diagnosis (or treatment initiation) until death from any cause. BCSS refers to the duration patients with breast cancer live post-diagnosis without succumbing to cancer-related complications or progression (Figure 1).

### 2.2 Variable selection

The specific study variables included: age at diagnosis (70–74, 75–79, 80–84, and ≥85 years); race (white, black, other); marital status (married, unmarried); pathological type (infiltrating duct carcinoma, other); tumor location (left, right); American Joint Committee on Cancer (AJCC) T staging (T1, T2); AJCC N staging (N0, N1); surgical method (modified radical mastectomy, breast-conserving surgery); and chemotherapy status (yes, none).

### 2.3 Statistical analyses

Baseline characteristics were compared using the chi-square test. We employed propensity score matching (PSM) with a caliper of 0.001 to eliminate intergroup differences. PSM could reduce selection bias by matching individuals with similar propensity scores, resulting in more comparable study groups and enhancing the validity of comparisons (23). Univariate and multivariate Cox regression analyses identified independent risk factors in different populations, followed by subgroup analysis of the patients. A nomogram model was then constructed based on the independent risk factors. To evaluate the model's discriminative ability, the receiver operating characteristic (ROC) curve was plotted, along with the AUC and concordance index (C-index). The calibration plot evaluated whether the expected outcomes matched the actual findings. Then, the population was scored based on the nomogram, classifying patients into low-score, and high-score groups using X-tile software. Kaplan-Meier (KM) survival analysis was conducted to compare OS and BCSS among different cohorts. Statistical significance was assessed using a two-sided *p*-value of < 0.05 and data analysis was conducted using R software (version 4.4.1; http://www.R-project.org/).

## 3 Results

### 3.1 Baseline characteristics

Following strict adherence to our inclusion and exclusion criteria, a total of 3,024 elderly patients with early-stage TNBC were enrolled in this study. The population was divided into two groups based on the receipt of RT: the RT group (*N* = 2,045; 67.6%) and the non-radiotherapy group (*N* = 979; 32.4%). Our analysis revealed that patients in the RT group were generally younger (RT, ≥85 years, 8.9% vs. non-RT, ≥85 years, 21.5%) and presented with earlier T, N staging. Furthermore, most patients in this cohort underwent BCS (breast-conserving surgery; *N* = 1,945; 95.1%), whereas a large proportion, opted for chemotherapy compared to the non-RT group (RT, undergo chemotherapy, 47.6% vs. non-RT, undergo chemotherapy, 31.5%). It is clear that baseline characteristics between the two groups were unbalanced, prompting the use of PSM. After 1:1 matching with a caliper width of 0.001, 569 patient pairs were successfully matched. The covariates between the two groups were all below 0.05, indicating a robust balance that significantly mitigated confounding bias in the study. The baseline characteristics of the population before and after PSM are presented in Table 1.

**Table 1:** Demographic, clinical, and laboratory features of TNBC patients aged over 70 with T1-2N0-1M0 stage before and after PSM.

**Variables**	**Initial cohort**			**PSM cohort**		
	**RT**	**Non-RT**	***P***	**RT**	**Non-RT**	***P***
	***N*** = **2,045**	***N*** = **979**		***N*** = **569**	***N*** = **569**	
**Age (years)**			< 0.001			0.990
70–74	920 (45.0%)	325 (33.2%)		202 (35.5%)	207 (36.4%)	
75–79	584 (28.6%)	238 (24.3%)		147 (25.8%)	144 (25.3%)	
80–84	359 (17.6%)	206 (21.0%)		121 (21.3%)	119 (20.9%)	
85+	182 (8.9%)	210 (21.5%)		99 (17.4%)	99 (17.4%)	
**Race**			0.243			0.907
White	1,653 (80.8%)	766 (78.2%)		473 (83.1%)	469 (82.4%)	
Black	276 (13.5%)	148 (15.1%)		71 (12.5%)	72 (12.7%)	
Other	116 (5.7%)	65 (6.6%)		25 (4.4%)	28 (4.9%)	
**Marital status**			0.582			0.443
Married	894 (91.3%)	1,881 (92.0%)		533 (93.7%)	540 (94.9%)	
Unmarried	85 (8.7%)	164 (8.0%)		36 (6.3%)	29 (5.1%)	
**Laterality**						0.721
Left	1,074 (52.5%)	523 (53.4%)		301 (52.9%)	308 (54.1%)	
Right	971 (47.5%)	456 (46.6%)		268 (47.1%)	261 (45.9%)	
**Histopathology**			0.054			0.626
Duct carcinoma	1,735 (84.8%)	803 (82.0%)		475 (83.5%)	482 (84.7%)	
Other	310 (15.2%)	176 (18.0%)		94 (16.5%)	87 (15.3%)	
**Grade**			0.212			0.677
I–II	587 (28.7%)	259 (26.5%)		134 (23.6%)	141 (24.8%)	
III	1,458 (71.3%)	720 (73.5%)		435 (76.5%)	428 (75.2%)	
**T**			< 0.001			0.951
T1	1,378 (67.4%)	508 (51.9%)		342 (60.1%)	344 (60.5%)	
T2	667 (32.6%)	471 (48.1%)		227 (39.9%)	225 (39.5%)	
**N**			< 0.001			1.000
N0	1,716 (83.9%)	756 (77.2%)		468 (82.2%)	468 (82.2%)	
N1	329 (16.1%)	223 (22.8%)		101 (17.8%)	101 (17.8%)	
**Surgery**			< 0.001			1.000
BCS	1,945 (95.1%)	587 (60.0%)		503 (88.4%)	503 (88.4%)	
Mastectomy	100 (4.9%)	392 (40.0%)		66 (11.6%)	66 (11.6%)	
**Chemotherapy**			< 0.001			0.807
No/unknown	1,071 (52.4%)	671 (68.5%)		347 (61.0%)	352 (61.9%)	
Yes	974 (47.6%)	308 (31.5%)		222 (39.0%)	217 (38.1%)	

TNBC, triple-negative breast cancer; PSM, propensity score matching; BCS, breast-conserving surgery; RT, radiotherapy. For race, other includes American Indian, AK Native, Asian, and Pacific Islander.

### 3.2 Survival analysis of individuals following PSM and subgroup analysis in BCS group

**Figure 2:**
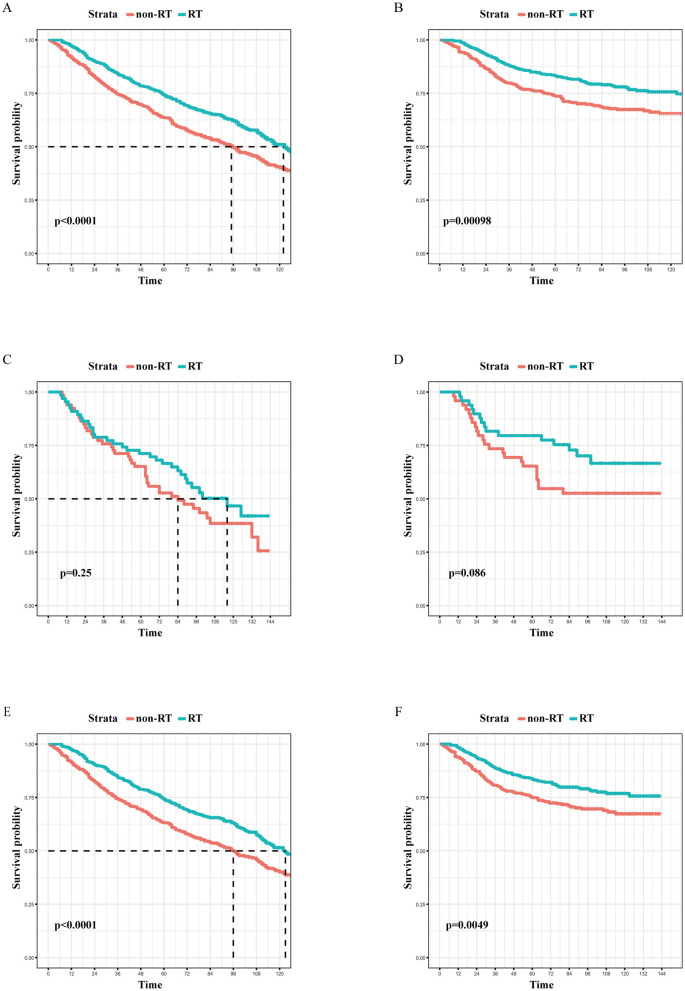
The Kaplan-Meier survival curves of the effect of RT on OS in the overall cohort **(A)**, the mastectomy cohort **(C)**, and the BCS cohort **(E)**, as well as the effect of RT on BCSS in the overall cohort **(B)**, the mastectomy cohort **(D)**, and the BCS cohort **(F)** after PSM. RT, radiotherapy; OS, overall survival; BCS, breast-conserving surgery; PSM, propensity score matching.

**Figure 3:**
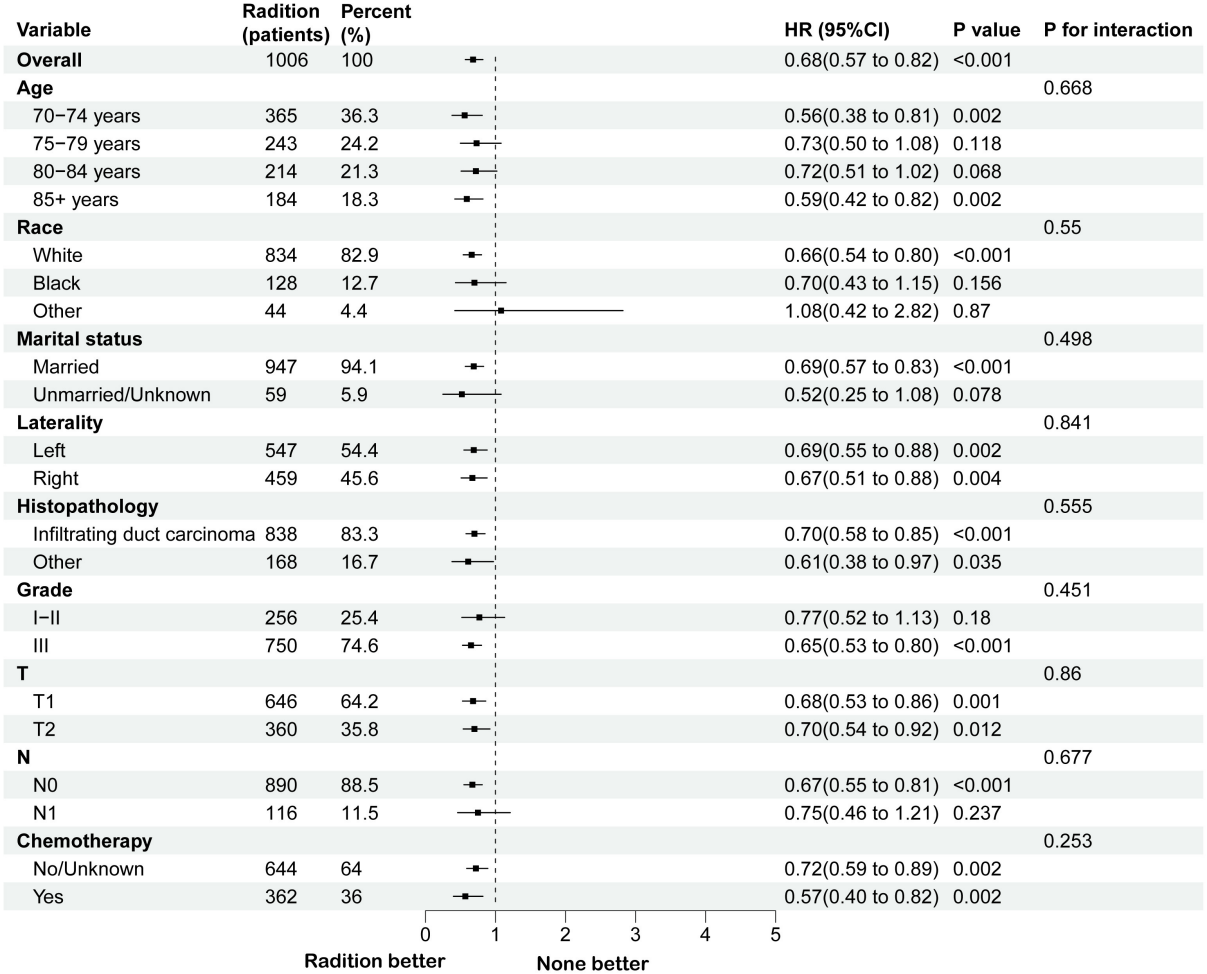
Subgroup analysis for OS between RT and no RT groups after PSM in BCS group. RT, radiotherapy; OS, overall survival; PSM, propensity score matching; BCS, breast-conserving surgery.

**Figure 4:**
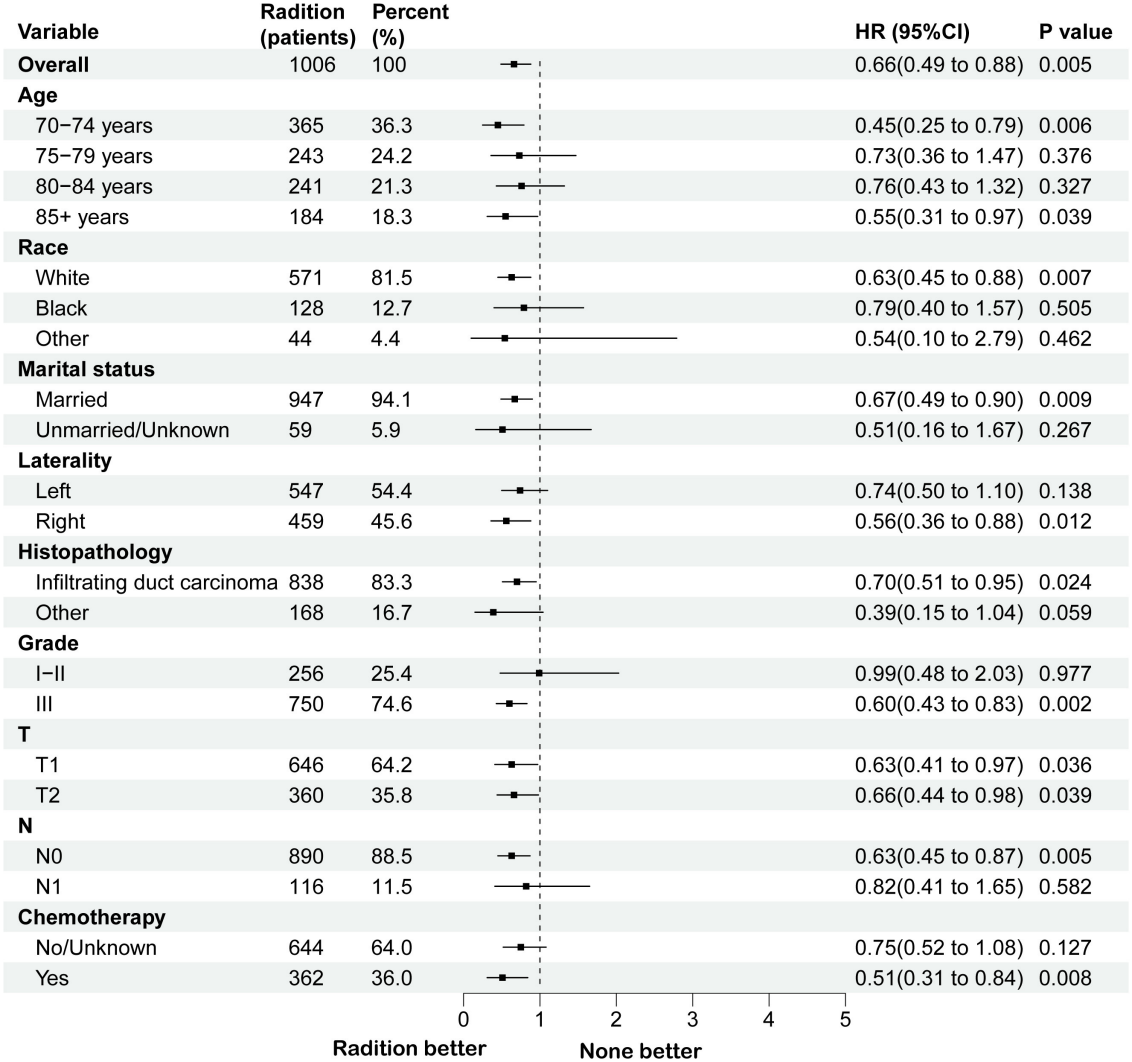
Subgroup analysis for BCSS between RT and no RT groups after PSM in BCS group. RT, radiotherapy; BCSS, breast cancer-specific survival; PSM, propensity score matching; BCS, breast-conserving surgery.

A survival analysis on the matched cohort was performed (Figure 2), revealing that the RT group experienced significantly greater survival benefits, as evidenced by both OS (HR = 0.69; 95% CI = 0.60–0.79; *p* < 0.001) and BCSS (HR = 0.64; 95% CI = 0.51–0.80; *p* = 0.001). Postoperative RT is typically regarded as a standard treatment modality following BCS. For patients who have undergone modified radical mastectomy with more than three lymph node metastases or tumors measuring 5 cm or greater, RT is generally recommended (24). Accordingly, we performed further survival analysis based on the type of surgical intervention. As illustrated in Figure 2, RT offers significant survival benefits for the BCS population, both in terms of OS (HR = 0.68; 95% CI = 0.58–0.79; *p* < 0.001) and BCSS (HR = 0.65; 95% CI = 0.51–0.84; *p* = 0.005). However, this benefit is not evident in the modified radical mastectomy group, where the *p*-values are >0.05. Then, subgroup analysis of OS and BCSS in the BCS group showed that, similar to previous studies, RT improved both OS and BCSS. It was statistically significant for OS in patients with age (70–74/>85 years), race (White), marital status (married), Grade (III), T stage (T1, T2), N stage (N0), and chemotherapy (yes/no). For BCSS, RT showed statistical significance in patients with age (70–74/>85 years), race (White), marital status (married), Grade (III), T stage (T1, T2), N stage (N0), and chemotherapy (yes; Figures 3, 4).

### 3.3 Establishment and validation of the nomogram model

**Figure 5:**
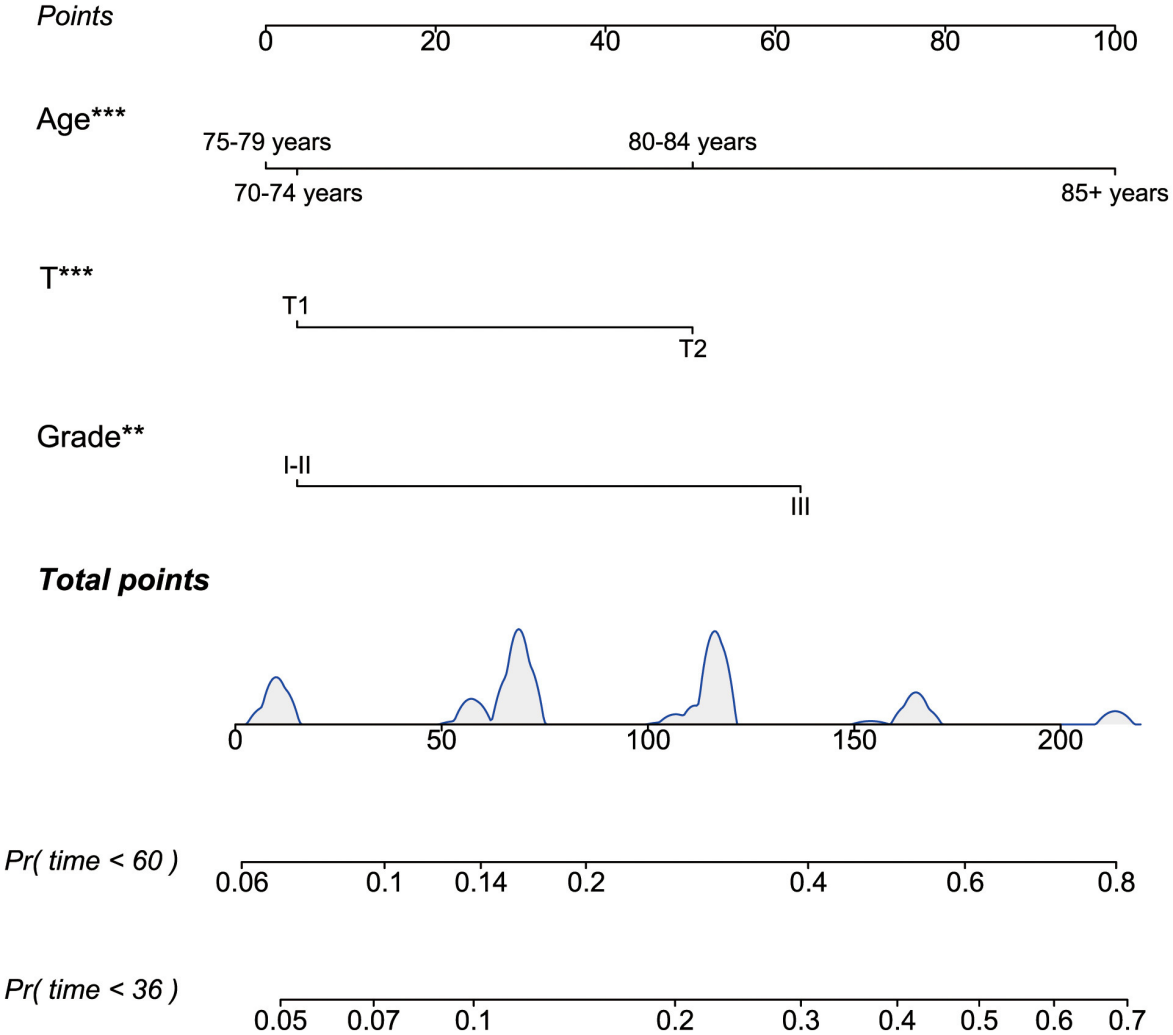
The nomogram to predict 3- and 5-year BCSS for the BCS cohort who did not receive RT after PSM. BCSS, breast cancer-specific survival; BCS, breast-conserving surgery; RT, radiotherapy; PSM, propensity score matching. ^**^P < 0.01; ^***^P < 0.001.

To identify independent risk factors associated with BCSS in patients from non-RT following BCS, both univariate and multivariate Cox regression analyses were performed on this cohort. Univariate and multivariate cox regression analysis identified age, tumor grade, and T stage as independent risk factors for BCSS, with older age, higher grade, and advanced T stage worsening prognosis (all *p*-values < 0.05). The *p*-values, hazard ratios (HR), and 95% confidence intervals (CI) for each independent parameter are presented in Table 2. Subsequently, utilizing the three independent risk factors identified, we developed a nomogram to predict 3- and 5-year BCSS for each patient and computed the scores associated with each risk factor (Figure 5).

**Table 2:** Univariate and multivariate Cox regression analyses of BCSS in patients from the BCS group who did not receive RT.

**Variables**	**Univariate analysis**		**Multivariate analysis**	
	**HR (95% CI)**	***P***	**HR (95% CI)**	***P***
**Age (years)**				
70–74	Reference		Reference	
75–79	0.84 (0.47–1.51)	0.581	0.94 (0.57–1.54)	0.849
80–84	1.88 (1.11–3.16)	0.017	2.04 (1.32–3.17)	0.007
85+	4.08 (2.22–6.89)	< 0.001	4.39 (2.82–6.83)	< 0.001
**Race**				
White	Reference		
Black	1.39 (0.89–2.16)	0.215		
Other	0.80 (0.37–1.72)	0.639		
**Marital status**				
Married	Reference		
Unmarried	1.33 (0.66–2.66)	0.497		
**Laterality**				
Left	Reference		
Right	0.96 (0.65–1.43)	0.873		
**Histopathology**				
Duct carcinoma	Reference		
Other	0.68 (0.37–1.24)	0.213		
**Grade**				
I–II	Reference		Reference	
III	2.62 (1.63–3.21)	< 0.001	2.48 (1.53–4.03)	0.002
**T**				
T1	Reference		Reference	
T2	2.46 (1.77–3.42)	< 0.001	2.04 (1.45–2.87)	< 0.001
**N**				
N0	Reference		
N1	1.62 (0.95–2.77)	0.074		
**Chemotherapy**				
No/unknown	Reference		
Yes	0.83 (0.59–1.16)	0.368		

BCSS, breast cancer-specific survival; BCS, breast-conserving surgery; RT, radiotherapy.

For race, other includes American Indian, AK Native, Asian, and Pacific Islander.

**Figure 6:**
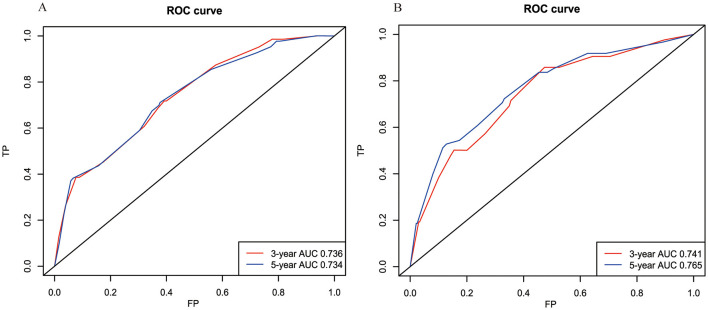
The receiver operating characteristic (ROC) curves for the BCSS model in the internal cohort **(A)** and external cohort **(B)**. BCSS, breast cancer-specific survival.

The model's reliability was assessed through ROC analysis, revealing AUCs of 0.736, and 0.734 for the 3- and 5-year BCSS nomogram, respectively. The C-index was determined to be 0.709. Given the constraints of sample size, patients from the RT group were utilized for external validation, yielding AUCs of 0.741 and 0.765 at 3 and 5 years, respectively, with a concordance index of 0.728 (Figure 6). Furthermore, the calibration curves for BCSS at 3 and 5 years in both the internal and external validation cohorts closely align with the standard curve (Supplementary Figure 1). This suggests that the model demonstrates robust reliability and effective discriminative capacity.

### 3.4 Analysis of survival in risk stratification groups

Based on the scores derived from the nomogram, specific scores were calculated for all patients in the RT and non-RT groups following PSM. Subsequently, the x-tile software was employed to classify all patients into low-risk (total nomogram score < 60, *N* = 316, 31.41%), intermediate-risk (total nomogram score ≥60 and < 100, *N* = 211, 20.97%) and high-risk groups (total nomogram score ≥100, *N* = 479, 47.61%).

It was found that RT is a crucial treatment modality for patients in the intermediate-risk (OS, HR = 0.49, 95% CI = 0.34–0.71, *p* = 0.001; BCSS, HR = 0.40, 95% CI = 0.21–0.77, *p* = 0.018) and high-risk group (OS, HR = 0.67, 95% CI = 0.55–0.81, *p* < 0.001; BCSS, HR = 0.61, 95% CI = 0.45–0.83, *p* = 0.007), whereas in the low-risk group, patients appear to be exempt from RT (OS, HR = 0.89, 95% CI = 0.65–1.22, *p* = 0.571; BCSS, HR = 0.91, 95% CI = 0.50–1.65, *p* = 0.806; Supplementary Figure 2).

## 4 Discussion

Aging is a significant risk factor for many chronic diseases, including cardiovascular diseases, cancer, and neurodegenerative disorders (25–28). Advances in medical technology have significantly extended the average lifespan of patients globally, which also presents new challenges for the treatment of the elderly population (29). While certain studies indicate that postoperative RT may enhance survival outcomes in early-stage BC patients (30, 31), the requirement for this treatment in elderly patients remains a subject of debate. Multiple comorbidities, reduced physiological function, and higher postoperative complications in older individuals may reduce RT effectiveness. These issues worsen with age (9, 32).

Due to the relatively poor prognosis of TNBC compared to other molecular subtypes (33), this study selected elderly women with early-stage TNBC from the SEER database to investigate the survival benefits of RT in this population, providing evidence for clinical decision-making.

In this study, we observed that RT offers substantial improvements in OS and BCSS among the population following PSM. In fact, the well-known CALGB 9343 study found that the local recurrence rate (LRR) in the RT group was 2% (95% CI, 96–99%), significantly lower than the 10% (95% CI, 85–93%) observed in the non-RT group. However, no significant difference in OS was identified between the two groups; the 10-year OS for the RT group was 67% (95% CI, 62–72%), compared to 66% (95% CI, 61–71%) for the non-RT group. In the follow-up PRIME II study, researchers concluded that postoperative RT after BCS led to a notable but moderate decrease in LRR over 5 years (14, 34). For some patients, the risk of ipsilateral breast tumor recurrence within this timeframe might be sufficiently low to justify the omission of RT (34). This finding appears to contradict our results. However, these two trials lacked comprehensive analysis of different molecular subtypes and tumor grades (14).

Given that the surgical approach is a crucial determinant of whether patients require postoperative RT (35–38), the population was categorized into the BCS group and the modified radical mastectomy group for a more detailed analysis. The results indicate that RT appears to provide more significant benefits for patients in the BCS cohort (OS, *p* < 0.001; BCSS, *p* = 0.005), while the effects are relatively modest for patients in the modified radical mastectomy group (OS, *p* = 0.25; BCSS, *p* = 0.086). This may be due to more complete cancer removal through modified radical mastectomy, which also increases surgical trauma and complications (39). However, it is important to recognize that a subset of the elderly population can benefit from postoperative RT (40, 41). Therefore, a Cox regression analysis was performed on the BCS group without RT, and a nomogram model for BCSS was developed based on independent risk factors. Although earlier studies used LRR to assess postoperative RT in BCS, the SEER database lacked relevant data. The predictive model focused on BCSS, which indirectly indicates LRR. It also helps predict OS in stratified analyses (42, 43). According to our nomogram model, an increase in tumor aggressiveness factors (such as T stage and Grade) and patient age are typically associated with a poorer prognosis. Therefore, when scoring based on patient risk levels, the low-risk group predominantly consists of younger individuals with smaller tumor sizes and lower grade classifications. Survival analysis revealed that RT provided no survival benefit for the low-risk group, regardless of OS (HR = 0.89, 95% CI = 0.65–1.22, *p* = 0.571) or BCSS (HR = 0.91, 95% CI = 0.50–1.65, *p* = 0.806). The same analysis was conducted for the intermediate-risk and high-risk groups. Surprisingly, the intermediate- (OS, HR = 0.49, 95% CI = 0.34–0.71, *p* = 0.001; BCSS, HR = 0.40, 95% CI = 0.21–0.77, *p* = 0.019) and high-risk groups (OS, HR = 0.67, 95% CI = 0.55–0.81, *p* < 0.001; BCSS, HR = 0.61, 95% CI = 0.45–0.83, *p* = 0.007) —demonstrated significant improvements in prognosis following RT, indicating benefits for both OS and BCSS. This suggests that in clinical management, low-risk patients may be exempt from RT based on individual characteristics, while patients with medium to high risk are recommended to undergo appropriate postoperative RT to enhance prognosis.

Therefore, in clinical practice, the treatment of elderly patients should not follow a one-size-fits-all approach. Based on our findings, patients in intermediate and high-risk groups may benefit from RT. A thorough evaluation of factors such as age, grade, and T stage is required to identify those likely to benefit from RT. This approach aims to personalize treatment based on individual risk profiles, in contrast to the generalized notion that conservative treatment is usually preferable for elderly patients. Our findings may provide new treatment strategies for elderly patients with early-stage TNBC and establish a model for BCS populations, emphasizing the importance of personalized treatment. Overall, this study is the first to examine the impact of postoperative RT in patients over 70 years old with T1-2N0-1M0 stage TNBC. Using PSM to adjust for confounders, significant survival differences were observed in the BCS group. Cox regression analysis of BCSS led to the development of nomogram and calibration curves, with AUC and C-index both >0.7, indicating strong predictive ability. As noted, due to the current lack of studies focused on elderly BC patients, this research offers valuable insights for improving clinical decision-making and providing more precise treatment for elderly BC patients. Based on our conclusions, we strongly recommend postoperative RT as an adjuvant treatment for patients in the intermediate and high-risk groups. Conversely, we do not recommend postoperative RT for low-risk patients. However, in clinical practice, a comprehensive assessment of the patient's overall health status and comorbidities should be conducted to inform treatment decisions.

The study has several limitations. First, the SEER database lacks specific chemotherapy regimens and detailed radiotherapy dosages. Consequently, despite utilizing PSM, potential confounding factors may still influence the results. Second, although the database was used as the data source, the sample size remains relatively small; the nomogram model is based on only 330 patients with BCSS, which may introduce bias. Future research should utilize real-world data to comprehensively address these issues and validate the findings in a real-world context. In conclusion, this study aims to address the ambiguity surrounding radiotherapy indications for elderly patients, improve current treatment protocols, and provide survival benefits for the elderly TNBC population.

## 5 Conclusions

RT markedly improves OS and BCSS in early-stage TNBC patients after undergoing BCS. For those identified as medium to high-risk groups, RT is a critical element of cancer management and is recommended whenever physically feasible. In contrast, low-risk early-stage TNBC patients may be considered for omitting RT.

## Data Availability

The datasets presented in this study can be found in online repositories. The names of the repository/repositories and accession number(s) can be found in the article/Supplementary material.

## Supplementary material

Supplemental Figure 1 Calibration curves for predicting patients' BCSS at 3- and 5- years in the internal cohort **(A, B)** and external cohorts **(C, D)**. BCSS, breast cancer-specific survival.

Supplemental Figure 2 The Kaplan-Meier survival curves of the effect of RT on OS in the low-risk cohort **(A)**, intermediate-risk cohort **(C)**, and the high-risk cohort **(E)**, as well as the effect of RT on BCSS in the low-risk cohort **(B)**, intermediate-risk cohort **(D)**, and the high-risk cohort **(F)**. RT, radiotherapy; OS, overall survival; BCSS, breast cancer-specific survival.

## References

[1] Siegel RL Miller KD Wagle NS Jemal A. Cancer statistics, 2023. CA Cancer J Clin. (2023) 73:17–48. 10.3322/caac.21763 36633525

[2] Singh D Vignat J Lorenzoni V Eslahi M Ginsburg O Lauby-Secretan B. Global estimates of incidence and mortality of cervical cancer in 2020: a baseline analysis of the WHO Global Cervical Cancer Elimination Initiative. Lancet Glob Health. (2023) 11:e197–206. 10.1016/S2214-109X(22)00501-0 36528031 PMC9848409

[3] Muss HB Berry DA Cirrincione CT Theodoulou M Mauer AM Kornblith AB. Adjuvant chemotherapy in older women with early-stage breast cancer. N Engl J Med. (2009) 360:2055–65. 10.1056/NEJMoa0810266 19439741 PMC3082436

[4] Giugliano FM Falivene S Esposito E Di Franco R Muto M D'Aiuto M. External radiotherapy for breast cancer in the elderly. Aging Clin Exp Res. (2017) 29:149–57. 10.1007/s40520-016-0655-x 27837457

[5] DeSantis CE Fedewa SA Goding Sauer A Kramer JL Smith RA Jemal A. Breast cancer statistics, 2015: convergence of incidence rates between black and white women. CA Cancer J Clin. (2016) 66:31–42. 10.3322/caac.21320 26513636

[6] Caswell-Jin JL Sun LP Munoz D Lu Y Li Y Huang H. Analysis of breast cancer mortality in the US-1975 to 2019. J Am Med Assoc. (2024) 331:233–41. 10.1001/jama.2023.25881 38227031 PMC10792466

[7] Jatoi I Chen BE Anderson WF Rosenberg PS. Breast cancer mortality trends in the United States according to estrogen receptor status and age at diagnosis. J Clin Oncol. (2007) 25:1683–90. 10.1200/JCO.2006.09.2106 17404367

[8] Yasmeen F Hyndman RJ Erbas B. Forecasting age-related changes in breast cancer mortality among white and black US women: a functional data approach. Cancer Epidemiol. (2010) 34:542–9. 10.1016/j.canep.2010.05.001 20887940

[9] de Glas NA Kiderlen M Bastiaannet E de Craen AJM van de Water W van de Velde CJH. Postoperative complications and survival of elderly breast cancer patients: a FOCUS study analysis. Breast Cancer Res Treat. (2013) 138:561–9. 10.1007/s10549-013-2462-9 23446810

[10] Yuan C Xie Z Bian J Huo J Daily K. Outcomes of primary endocrine therapy in elderly women with stage I–III breast cancer: a SEER database analysis. Breast Cancer Res Treat. (2020) 180:819–27. 10.1007/s10549-020-05591-9 32172303

[11] Dimitrakopoulos FI Kottorou A Antonacopoulou AG Makatsoris T Kalofonos HP. Early-stage breast cancer in the elderly: confronting an old clinical problem. J Breast Cancer. (2015) 18:207–17. 10.4048/jbc.2015.18.3.207 26472970 PMC4600684

[12] Gosain R Pollock Y Jain D. Age-related disparity: breast cancer in the elderly. Curr Oncol Rep. (2016) 18:69. 10.1007/s11912-016-0551-8 27807821

[13] Gironés R Torregrosa D Díaz-Beveridge R. Comorbidity, disability and geriatric syndromes in elderly breast cancer survivors. Results of a single-center experience. Crit Rev Oncol. (2010) 73:236–45. 10.1016/j.critrevonc.2009.08.002 19748793

[14] Hughes KS Schnaper LA Bellon JR Cirrincione CT Berry DA McCormick B. Lumpectomy plus tamoxifen with or without irradiation in women age 70 years or older with early breast cancer: long-term follow-up of CALGB 9343. J Clin Oncol. (2013) 31:2382–7. 10.1200/JCO.2012.45.2615 23690420 PMC3691356

[15] Speers C Pierce LJ. Postoperative radiotherapy after breast-conserving surgery for early-stage breast cancer: a review. J Am Med Assoc Oncol. (2016) 2:1075–82. 10.1001/jamaoncol.2015.5805 27243924

[16] van de Water W Markopoulos C van de Velde CJ Seynaeve C Hasenburg A Rea D. Association between age at diagnosis and disease-specific mortality among postmenopausal women with hormone receptor-positive breast cancer. J Am Med Assoc. (2012) 307:590–7. 10.1001/jama.2012.84 22318280

[17] Bastiaannet E Liefers GJ de Craen AJ Kuppen PJ van de Water W Portielje JE. Breast cancer in elderly compared to younger patients in the Netherlands: stage at diagnosis, treatment and survival in 127,805 unselected patients. Breast Cancer Res Treat. (2010) 124:801–7. 10.1007/s10549-010-0898-8 20428937

[18] Pötter R Gnant M Kwasny W Tausch C Handl-Zeller L Pakisch B. Lumpectomy plus tamoxifen or anastrozole with or without whole breast irradiation in women with favorable early breast cancer. Int J Radiat Oncol Biol Phys. (2007) 68:334–40. 10.1016/j.ijrobp.2006.12.045 17363187

[19] Garrido-Castro AC Lin NU Polyak K. Insights into molecular classifications of triple-negative breast cancer: improving patient selection for treatment. Cancer Discov. (2019) 9:176–98. 10.1158/2159-8290.CD-18-1177 30679171 PMC6387871

[20] Dent R Trudeau M Pritchard KI Hanna WM Kahn HK Sawka CA. Triple-negative breast cancer: clinical features and patterns of recurrence. Clin Cancer Res. (2007) 13:4429–34. 10.1158/1078-0432.CCR-06-3045 17671126

[21] Hallett RM Dvorkin-Gheva A Bane A Hassell JA. A gene signature for predicting outcome in patients with basal-like breast cancer. Sci Rep. (2012) 2:227. 10.1038/srep00227 22355741 PMC3259129

[22] Che WQ Li YJ Tsang CK Wang YJ Chen Z Wang XY. How to use the Surveillance, Epidemiology, and End Results (SEER) data: research design and methodology. Milit Med Res. (2023) 10:50. 10.1186/s40779-023-00488-2 37899480 PMC10614369

[23] Chen JW Maldonado DR Kowalski BL Miecznikowski KB Kyin C Gornbein JA. Best practice guidelines for propensity score methods in medical research: consideration on theory, implementation, and reporting. A review. Arthroscopy. (2022) 38:632–42. 10.1016/j.arthro.2021.06.037 34547404

[24] Offersen BV Overgaard M Overgaard J. Breast cancer radiotherapy: is it time to rethink the indication? Radiother Oncol. (2022) 177:238–9. 10.1016/j.radonc.2022.10.009 36265681

[25] Cuenca-Bermejo L Prinetti A Kublickiene K Raparelli V Kautzky-Willer A Norris CM. Fundamental neurochemistry review: old brain stories - Influence of age and sex on the neurodegeneration-associated lipid changes. J Neurochem. (2023) 166:427–52. 10.1111/jnc.15834 37161795

[26] Kennedy BK Berger SL Brunet A Campisi J Cuervo AM Epel ES. Geroscience: linking aging to chronic disease. Cell. (2014) 159:709–13. 10.1016/j.cell.2014.10.039 25417146 PMC4852871

[27] Makino K Lee S Bae S Chiba I Harada K Katayama O. Absolute cardiovascular disease risk assessed in old age predicts disability and mortality: a retrospective cohort study of community-dwelling older adults. J Am Heart Assoc. (2021) 10:e022004. 10.1161/JAHA.121.022004 34913358 PMC9075253

[28] Montégut L López-Otín C Kroemer G. Aging and cancer. Mol Cancer. (2024) 23:106. 10.1186/s12943-024-02020-z 38760832 PMC11102267

[29] Cai Y Song W Li J Jing Y Liang C Zhang L. The landscape of aging. Sci China Life Sci. (2022) 65:2354–454. 10.1007/s11427-022-2161-3 36066811 PMC9446657

[30] Hung SK Yang HJ Lee MS Liu DW Chen LC Chew CH. Molecular subtypes of breast cancer predicting clinical benefits of radiotherapy after breast-conserving surgery: a propensity-score-matched cohort study. Breast Cancer Res. (2023) 25:149. 10.1186/s13058-023-01747-9 38066611 PMC10709935

[31] Xia LY Xu WY Zhao Y. Effect of postmastectomy radiotherapy on T1-2N1M0 triple-negative breast cancer. PLoS ONE. (2022) 17:e0270528. 10.1371/journal.pone.0270528 35749525 PMC9231765

[32] Darby S McGale P Correa C Taylor C Arriagada R Clarke M. Effect of radiotherapy after breast-conserving surgery on 10-year recurrence and 15-year breast cancer death: meta-analysis of individual patient data for 10,801 women in 17 randomised trials. Lancet. (2011) 378:1707–16. 10.1016/S0140-6736(11)61629-2 22019144 PMC3254252

[33] Bai X Ni J Beretov J Graham P Li Y. Triple-negative breast cancer therapeutic resistance: where is the Achilles' heel? Cancer Lett. (2021) 497:100–11. 10.1016/j.canlet.2020.10.016 33069769

[34] Kunkler IH Williams LJ Jack WJ Cameron DA Dixon JM. Breast-conserving surgery with or without irradiation in women aged 65 years or older with early breast cancer (PRIME II): a randomised controlled trial. Lancet Oncol. (2015) 16:266–73. 10.1016/S1470-2045(14)71221-5 25637340

[35] Castaneda SA Strasser J. Updates in the treatment of breast cancer with radiotherapy. Surg Oncol Clin N Am. (2017) 26:371–82. 10.1016/j.soc.2017.01.013 28576177

[36] Dewar JA. Postmastectomy radiotherapy. Clin Oncol. (2006) 18:185–90. 10.1016/j.clon.2005.11.006 16605049

[37] Kim H Lee SB Nam SJ Lee ES Park BW Park HY. Survival of breast-conserving surgery plus radiotherapy versus total mastectomy in early breast cancer. Ann Surg Oncol. (2021) 28:5039–47. 10.1245/s10434-021-09591-x 33492542

[38] Kolárová I Melichar B Sirák I Vanásek J Petera J Horáčková K. The role of adjuvant radiotherapy in the treatment of breast cancer. Curr Oncol. (2024) 31:1207–20. 10.3390/curroncol31030090 38534923 PMC10969207

[39] Kovačević P Miljković S Višnjić A Kozarski J Janković R. Quality of life indicators in patients operated on for breast cancer in relation to the type of surgery-a retrospective cohort study of women in Serbia. Medicina. (2020) 56:80402. 10.3390/medicina56080402 32796629 PMC7466215

[40] Kummerow KL Du L Penson DF Shyr Y Hooks MA. Nationwide trends in mastectomy for early-stage breast cancer. J Am Med Assoc Surg. (2015) 150:9–16. 10.1001/jamasurg.2014.2895 25408966

[41] McGuire KP Santillan AA Kaur P Meade T Parbhoo J Mathias M. Are mastectomies on the rise? a 13-year trend analysis of the selection of mastectomy versus breast conservation therapy in 5865 patients. Ann Surg Oncol. (2009) 16:2682-90. 10.1245/s10434-009-0635-x 19653046

[42] Johnson ME Handorf EA Martin JM Hayes SB. Postmastectomy radiation therapy for T3N0: a SEER analysis. Cancer. (2014) 120:3569–74. 10.1002/cncr.28865 24985911 PMC4413466

[43] Yang H Qiu M Feng Y Wen N Zhou J Qin X. The role of radiotherapy in HER2+ early-stage breast cancer patients after breast-conserving surgery. Front Oncol. (2022) 12:903001. 10.3389/fonc.2022.903001 36686782 PMC9845557

