# Supplementary figures and images for: The potential benefits of radiotherapy in elderly patients with early-stage triple-negative breast cancer
Source: Front Med (Lausanne). 2025 Jan 8;11:1525425. doi: 10.3389/fmed.2024.1525425 (PMC11751052; doi:10.3389/fmed.2024.1525425)

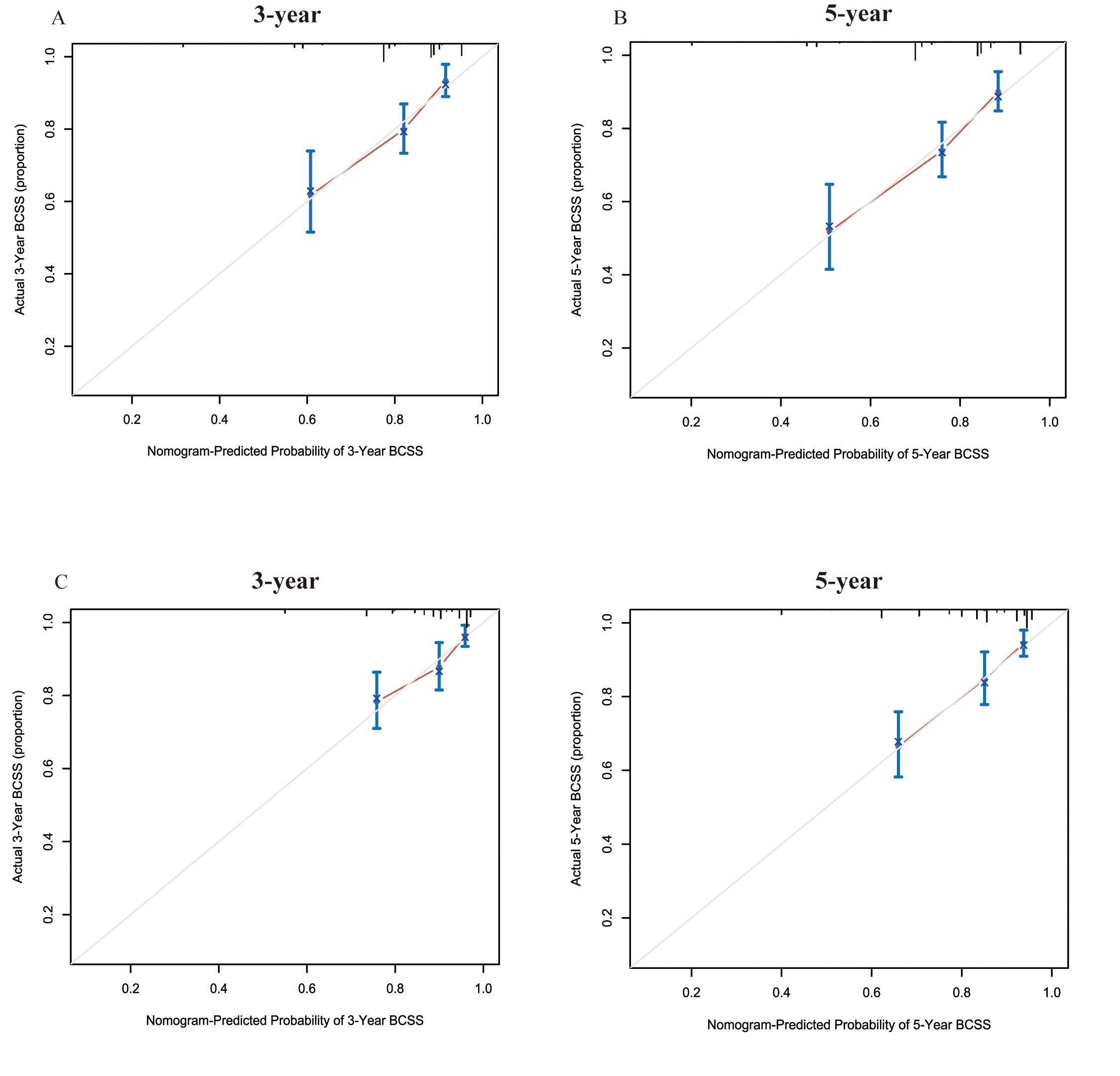

Supplement: Supplemental Figure 1 — Calibration curves for predicting patients' BCSS at 3- and 5- years in the internal cohort (A, B) and external cohorts (C, D). BCSS, breast cancer-specific survival. [file Image_1.tif]

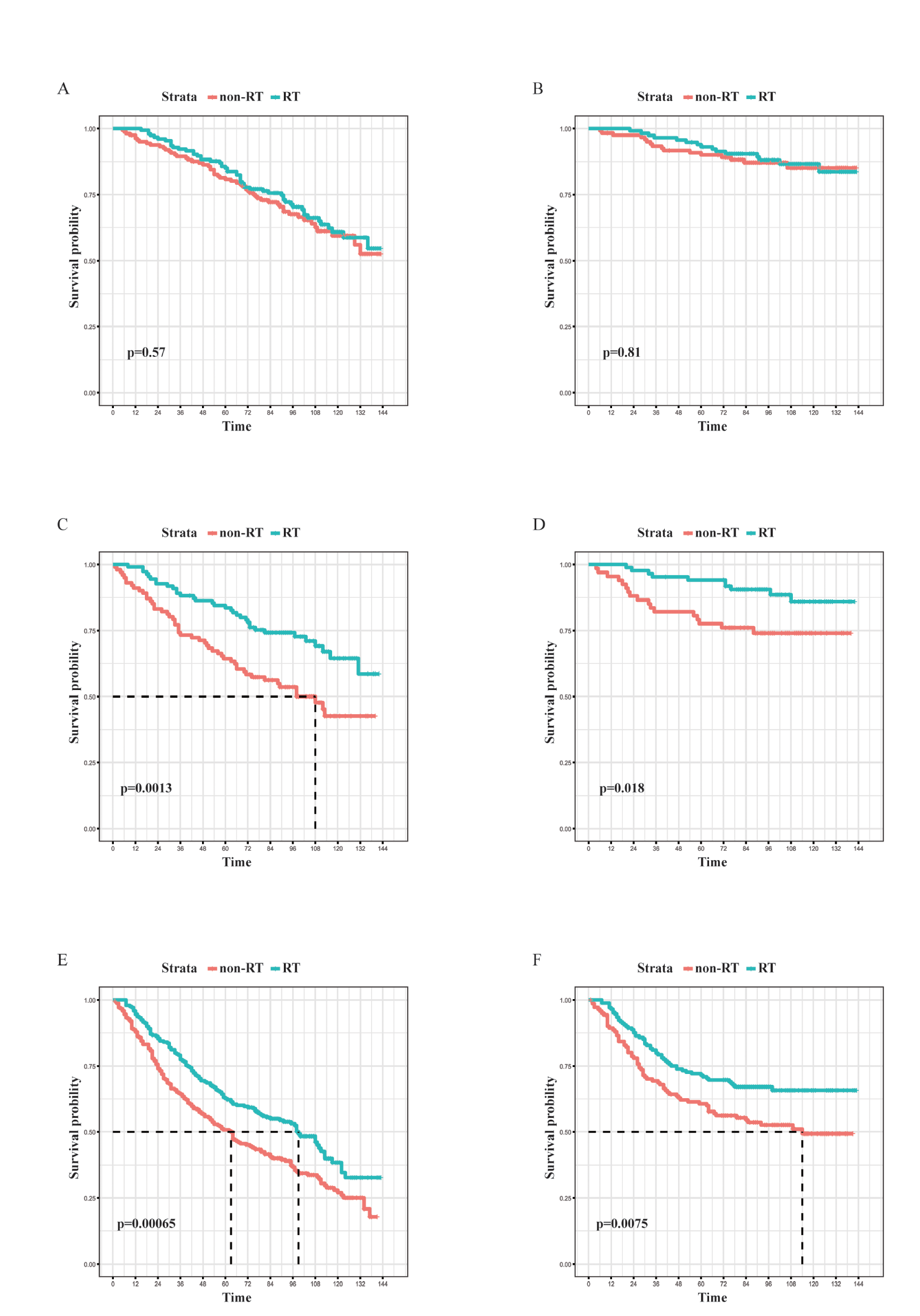

Supplement: Supplemental Figure 2 — The Kaplan-Meier survival curves of the effect of RT on OS in the low-risk cohort (A), intermediate-risk cohort (C), and the high-risk cohort (E), as well as the effect of RT on BCSS in the low-risk cohort (B), intermediate-risk cohort (D), and the high-risk cohort (F). RT, radiotherapy; OS, overall survival; BCSS, breast cancer-specific survival. [file Image_2.tif]
